# Sulfonium Salts as Acceptors in Electron Donor‐Acceptor Complexes

**DOI:** 10.1002/anie.202303104

**Published:** 2023-05-03

**Authors:** Leendert van Dalsen, Rachel E. Brown, James A. Rossi‐Ashton, David J. Procter

**Affiliations:** ^1^ Department of Chemistry The University of Manchester Manchester UK

**Keywords:** Aryl Radical, Arylation, Charge Transfer, EDA Complex, Sulfonium Salt

## Abstract

The photoactivation of electron donor‐acceptor complexes has emerged as a sustainable, selective and versatile strategy for the generation of radical species. Electron donor‐acceptor (EDA) complexation, however, imposes electronic constraints on the donor and acceptor components and this can limit the range of radicals that can be generated using the approach. New EDA complexation strategies exploiting sulfonium salts allow radicals to be generated from native functionality. For example, aryl sulfonium salts, formed by the activation of arenes, can serve as the acceptor components in EDA complexes due to their electron‐deficient nature. This “sulfonium tag” approach relaxes the electronic constraints on the parent substrate and dramatically expands the range of radicals that can be generated using EDA complexation. In this review, these new applications of sulfonium salts will be introduced and the areas of chemical space rendered accessible through this innovation will be highlighted.

## Graphical abstract

Photoactivation of electron donor‐acceptor (EDA) complexes is a sustainable, selective and versatile strategy for the generation of radicals. Sulfonium salt acceptors promise to dramatically expand the range of radicals that can be generated and exploited using EDA chemistry; the use of readily installed “sulfonium tags” relaxes the electronic constraints typically imposed on the parent substrate by an EDA complexation approach.

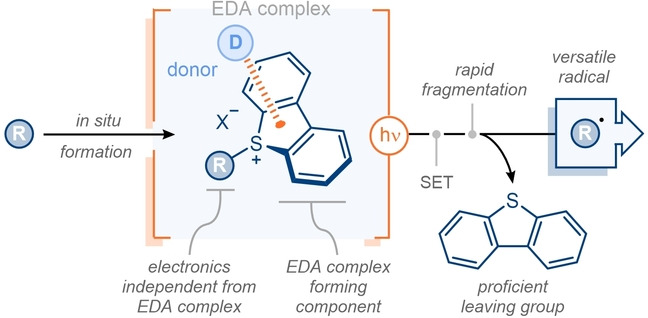

## 1 Introduction

Radicals, historically considered chaotic and uncontrollable, are now indispensable in synthetic chemistry. Their highly reactive nature enables reactivity that is otherwise difficult or impossible to achieve by other means and that is often complementary to polar or two‐electron manifolds.^[1]^

**Scheme 1:**
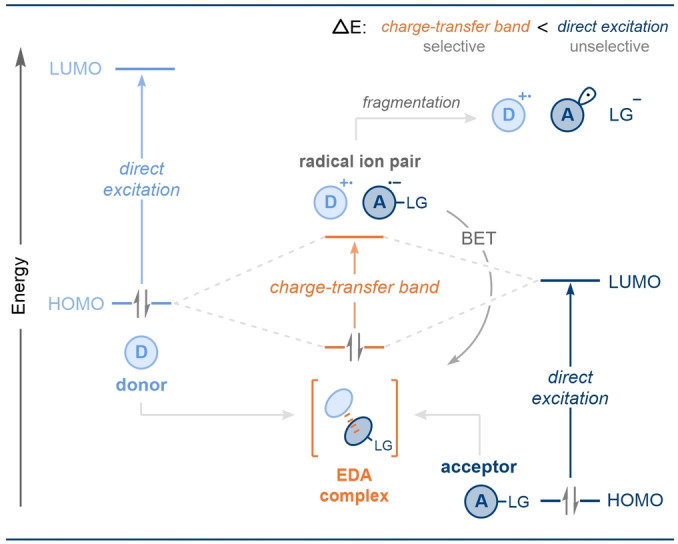
General molecular orbital comparison between direct photoexcitation and the excitation of an EDA complex.

Traditional photochemical radical generation employed direct photoexcitation, often requiring high energy (UV, ultraviolet) radiation to deliver two radical species by bond homolysis (Scheme 1). However, at these wavelengths many organic functionalities can also absorb light and thus react, rendering these processes uncontrolled, unselective, and ultimately leading to unsatisfactory product formation. As a result, the potential of synthetic photo‐mediated radical chemistry remained largely unfulfilled. In recent years, however, this situation has changed dramatically with the introduction of milder, and thus more selective, methods of radical generation that have greatly expanded the synthetic toolbox of chemists.^[2]^ Many such methods require the addition of exogenous photocatalysts,^[3]^ however, strategies that do not require photocatalysts are also attracting significant attention.

Photocatalyst‐free strategies typically exploit electron donor‐acceptor (EDA) complexes (also termed “charge‐transfer complexes”).^[4]^ EDA complexes result from the ground state association of an electron‐rich (donor) molecule with an electron‐poor (acceptor) molecule to form a molecular aggregate. In this complex a new electronic transition is possible—the charge transfer band—allowing the complex to absorb at a lower energy of light, typically in the visible region. Light irradiation at this wavelength can trigger a single‐electron‐transfer (SET) event generating a radical ion pair under far milder conditions with respect to radical generation by direct homolysis. Finally, the radical ion pair can follow different reaction pathways; for example, they can react with one another or they can undergo cage escape and react with external radical traps (also termed somophiles), such as unsaturated species. While light mediated SET is by far the most widely reported for EDA complexes, the SET event can also be triggered by thermal excitation.^[5]^

Although the photophysics of EDA complexes has been studied extensively since the 1950s,^[6]^ only recently have such complexes found application in synthetic chemistry. This can be accredited to the kinetically competitive rate of back electron transfer (BET) from the radical ion pair; BET simply reforms the ground state EDA components rather than furnishing synthetically useful radical species. To overcome BET, a suitable leaving group can be installed in either the acceptor or donor; upon SET, rapid and irreversible fragmentation, and loss of the leaving group, can outcompete BET and deliver reactive radical intermediates for productive chemistry.^[7]^ While the leaving group can be located on the donor—for example, DHPs (dihydropyridines),^[8]^ BF_3_K salts, and silicate salts,^[9]^ it is more commonly located on the acceptor—for example, organic halides,^[10]^ phenolates,^[11]^ and phthalimides.^[12]^ As a result, numerous successful EDA complex‐mediated radical processes have been developed and have been reviewed elsewhere.[^4c^, ^6d^, ^13^] This minireview instead focuses on the emergence of sulfonium salts as a new class of acceptor in synthetically useful EDA complexes.

Sulfonium salts are well established, typically bench stable compounds that can be readily formed with outstanding selectivity and then isolated or used in situ. They are defined as positively charged organo‐sulfur(IV) compounds in which the central sulfur atom is bonded to three organic substituents. They can undergo a great many transformations ranging from metal‐catalyzed and metal‐free cross‐coupling reactions to ylide reactivity, with their synthesis and established reactivity trends having been previously reviewed in detail.^[14]^

**Scheme 2:**
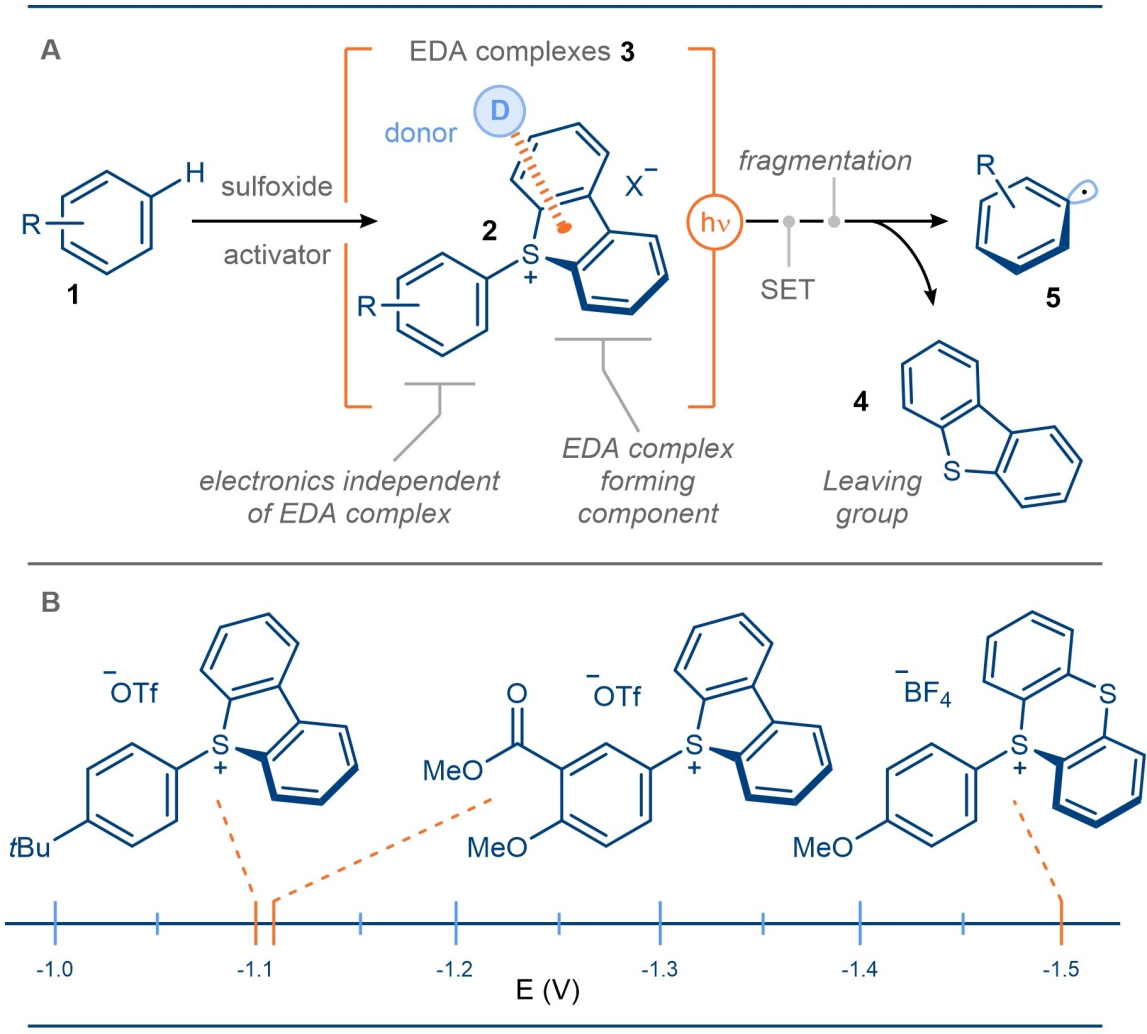
Sulfonium salt EDA complexation leading to radical release. An illustrative system involving aryl radical generation from in situ generated aryl sulfonium salts.

Early reports of sulfonium salts being used in EDA complexes are limited to trifluoromethyl radical generation from Umemoto's reagent^[15]^ and only recently has the utility of sulfonium salts truly been recognized. This is particularly the case for aryl radical generation (see section 2); easily derived sulfonium salts **2** form EDA complexes **3** with appropriate donor molecules. Upon excitation, a SET event triggers a fast, irreversible fragmentation in which sulfide **4** acts as a proficient leaving group, and desired radical **5** is formed, primed to undergo further reactivity (Scheme 2).

Importantly, beyond simply acting as a leaving group, such as with halides, the sulfur‐containing aromatic unit can serve as the electron‐accepting component, able to complex with the donor. To date, sulfur‐containing units possessing two linked aromatic groups—such as in dibenzothiophene (DBT), thianthrene (TT) and phenoxathiin—have been used to achieve complexation. Perhaps the most profound feature of this complex formation, is that the sulfur‐containing aromatic unit forms the EDA complex independent of the electronic nature of the rest of the acceptor molecule. Thus, parent arenes **1** no longer need to host electron‐withdrawing functionalities to lower their LUMOs and enable them to participate as acceptors in EDA complexes. This grants access to a far wider range of aryl radicals **5** using this platform. Such electronic independence is corroborated by the relatively consistent reduction potentials for aryl sulfonium salts bearing a range of substituents on the parent arene ring. In contrast, changes to the sulfur‐containing aromatic unit result in a more significant shift in the reduction potential.^[16]^ It is these key properties of sulfonium salt EDA complex formation that have recently been exploited in more general EDA complex‐mediated approaches for radical chemistry. Until processes can be developed that are catalytic in the sulfur component, it is important to note that, due to the highly UV active yet stable nature of the heterocyclic sulfide byproduct, it can be recovered with relative ease and efficiently recycled.

The goal of this review is to introduce sulfonium salts as versatile acceptors in EDA complexes and demonstrate how they have recently emerged as key substrates that allow some of the most significant challenges in EDA complex‐mediated radical generation to be overcome.

## 2 Aryl radical generation

Aryl radicals are proven versatile intermediates in synthetic chemistry. In particular, they are widely exploited in functional group interconversions and C−C bond‐forming reactions. However, due to their higher reactivity when compared to C(sp^3^) radicals, they are often considerably more challenging to form and control.^[17]^

EDA complexes have been used to generate aryl radicals, however, prior to the use of sulfonium salts as acceptors, the aryl radical precursors almost exclusively needed to contain electron‐withdrawing groups to successfully facilitate the formation of an EDA complex with a donor.[^10a^, ^10c^] Although *N*‐hydroxyphthalimide esters have the ability to form EDA complexes with donors independent of the electronics of the rest of the molecule[^12a^, ^18^]—much like sulfonium salts, the subsequent decarboxylation fragmentation event that forms the aryl radical is slow and undesirable pathways are observed.^[19]^ A further drawback of other EDA complex‐mediated aryl radical generation methods is the necessity for preinstalled halide or pseudo‐halide functionality to enable fragmentation upon SET. With aryl sulfonium salts, the now well‐established selective introduction of the sulfonium group at the expense of a C−H bond—including examples of late‐stage functionalization—and in situ use of the salts, negates the need for prefunctionalization.^[14a]^ As a result of these features, it is in aryl radical generation that sulfonium salt EDA acceptors have made the biggest impact to date.

### 2.1 C(sp^2^)−C bond‐forming reactions

**Scheme 3:**
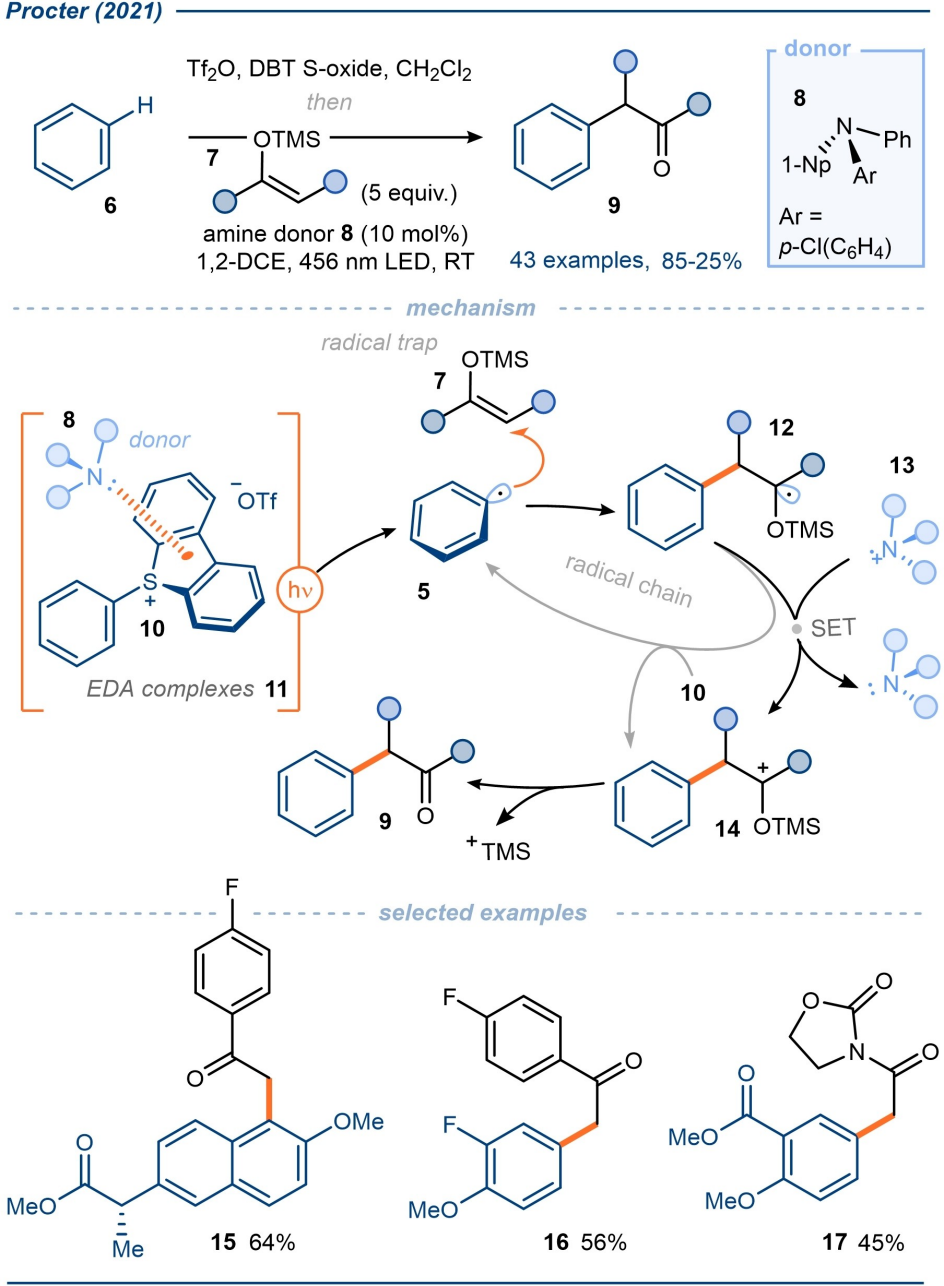
In situ formation and exploitation of aryl sulfonium salts in EDA complexes with triaryl amine donors; application in a formal C−H alkylation to give α‐aryl ketones.^[20]^ 1,2 DCE=1,2‐Dichloroethane, DBT S‐oxide=Dibenzothiophene *S*‐oxide, 1‐Np=1‐Naphthyl.

**Scheme 4:**
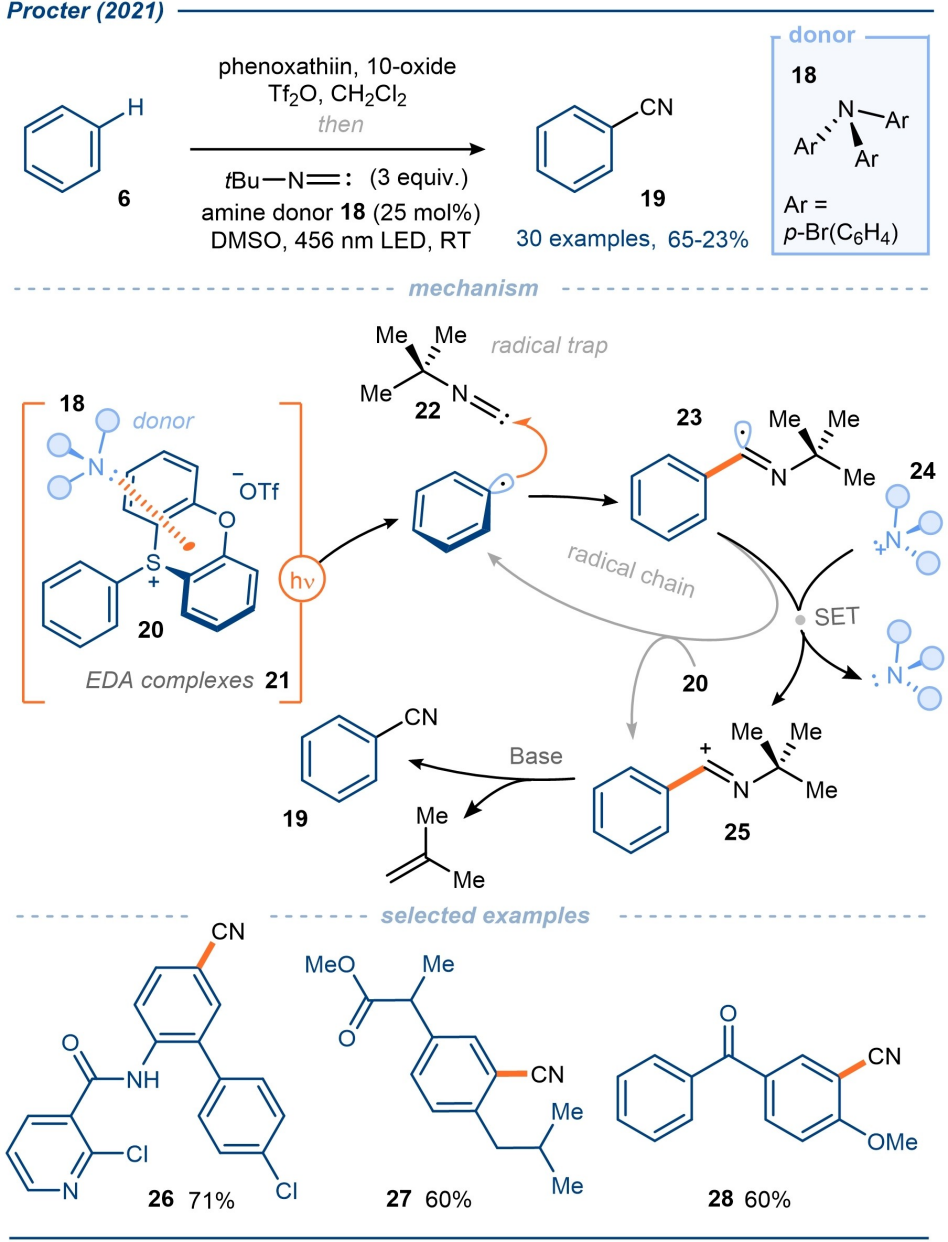
In situ formation and exploitation of aryl sulfonium salts in EDA complexes with triaryl amine donors; application in a formal C−H cyanation.^[20]^

In 2021, Procter and co‐workers first reported a general platform for the C−H functionalization of native arenes via EDA complex‐mediated aryl radical generation (Schemes 3 and 4).^[20]^ In a one‐pot process, parent arenes **6** underwent a regioselective interrupted Pummerer reaction with diaryl sulfoxides to give the corresponding triaryl sulfonium salts **10** and **20**—the mechanism of sulfonium salt formation has been previously reviewed in detail^[14e]^—which served as electron‐deficient acceptors in EDA complexes **11** and **21**. Key to this work was the identification of novel triarylamine donor molecule **8** (Scheme 3) and triarylamine donor **18** (Scheme 4), which were easily tunable and whose use reduced the reformation of the parent arene by HAT; the triarylamines do not possess any alpha‐amino hydrogen atoms. Under visible light irradiation, the resulting EDA complexes **11** and **21** generated aryl radicals which were then trapped by silyl enol ethers **7** resulting in formal C−H alkylation of the arene (Scheme 3), or by *tert*‐butyl isocyanide **22** to give products of formal C−H cyanation (Scheme 4).

A wide range of arenes were found to undergo C−H alkylation and cyanation, including electron‐rich aromatic systems, and complex bioactive scaffolds. Additionally, typically labile halides were also tolerated on the parent arene, underlining the greater propensity for reductive cleavage of the C−S bond vs the C−Hal bond; this selectivity could be manipulated to enable subsequent divergent coupling reactions, not possible with earlier EDA complex‐mediated aryl radical generation methods. The use of a catalytic amount of the amine donor **8** or **18**, suggests that a second SET event occurs from radical **12** or **23**, reducing the amine radical cation **13** or **24**, and regenerating the respective donor species in a closed cycle. Equally, in both alkylation and cyanation processes, a radical chain could be envisaged whereby the radical **12** or **23** directly reduce sulfonium salt **10** or **20** respectively, affording products **9** or **19**. That said, calculated quantum yields of 0.05 and 0.08 for the processes favor the operation of a closed cycle, however, a very weakly propagating radical chain can not be ruled out.

Typically, in C(sp^2^)−C bond‐forming reactions mediated by EDA complex photoactivation, the donor molecule also acts as the radical trap, thus limiting scope.[^10a^, ^21a^, ^21b^] In this work, however, with a separate donor and radical trap, a wider range of products is accessible. Furthermore, following the completion of each protocol (Scheme 3 and 4), recovery and reuse of the diaryl sulfide leaving group was demonstrated.

**Scheme 5:**
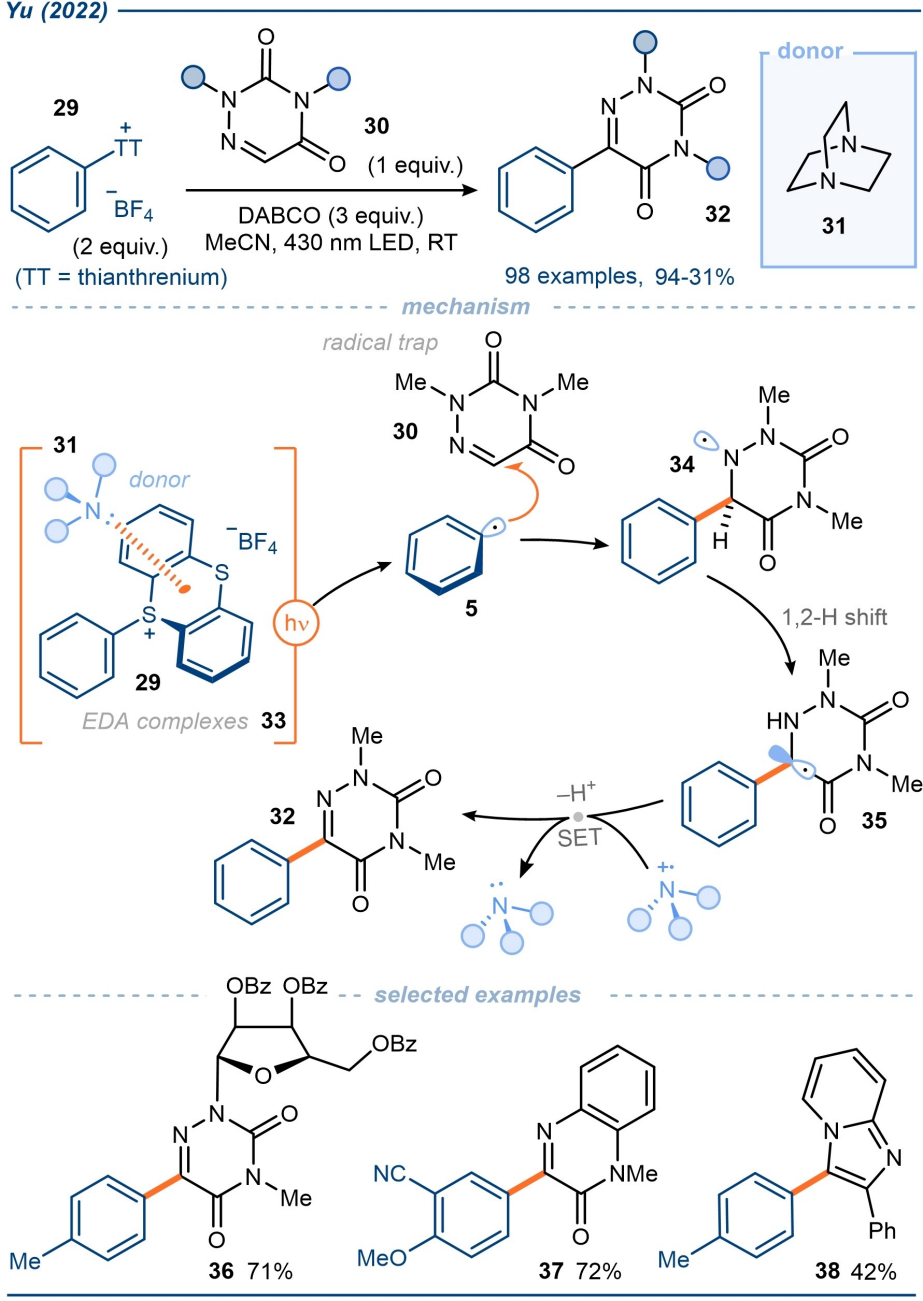
Exploitation of aryl sulfonium salts in EDA complexes with amine donors; application in aryl radical heteroarylation.^[22]^ DABCO=1,4‐Diazabicyclo[2.2.2]octane.

Yu and co‐workers also leveraged the selectivity of sulfonium salt formation to produce a range of thianthrenium salts **29** which were found to form EDA complexes with DABCO **31** (Scheme 5).^[22]^ The use of three equivalents of DABCO was found to be most effective while alternative commercially available amine donors led to sub‐optimal yields. The aryl radicals **5** generated by irradiation of the EDA complexes **33** were trapped by an impressive variety of densely functionalized unsaturated heterocyclic scaffolds, including a variety of azauracils **30**, nucleosides (to give **36**) and quinoxalinones (to give **37**). Mechanistically, the authors propose that the generated aryl radical **5**, adds to the unsaturated system **30** and, following a 1,2 H‐shift in the *N*‐centered radical **34**, a second SET step affords the arylated product **32**. To showcase the versatility of the process, the group also conducted the reaction using natural light, obtaining similar yields. Furthermore, some products were found to exhibit excellent antitumor activity.

### 2.2 C(sp^2^)−S bond‐forming reactions

**Scheme 6:**
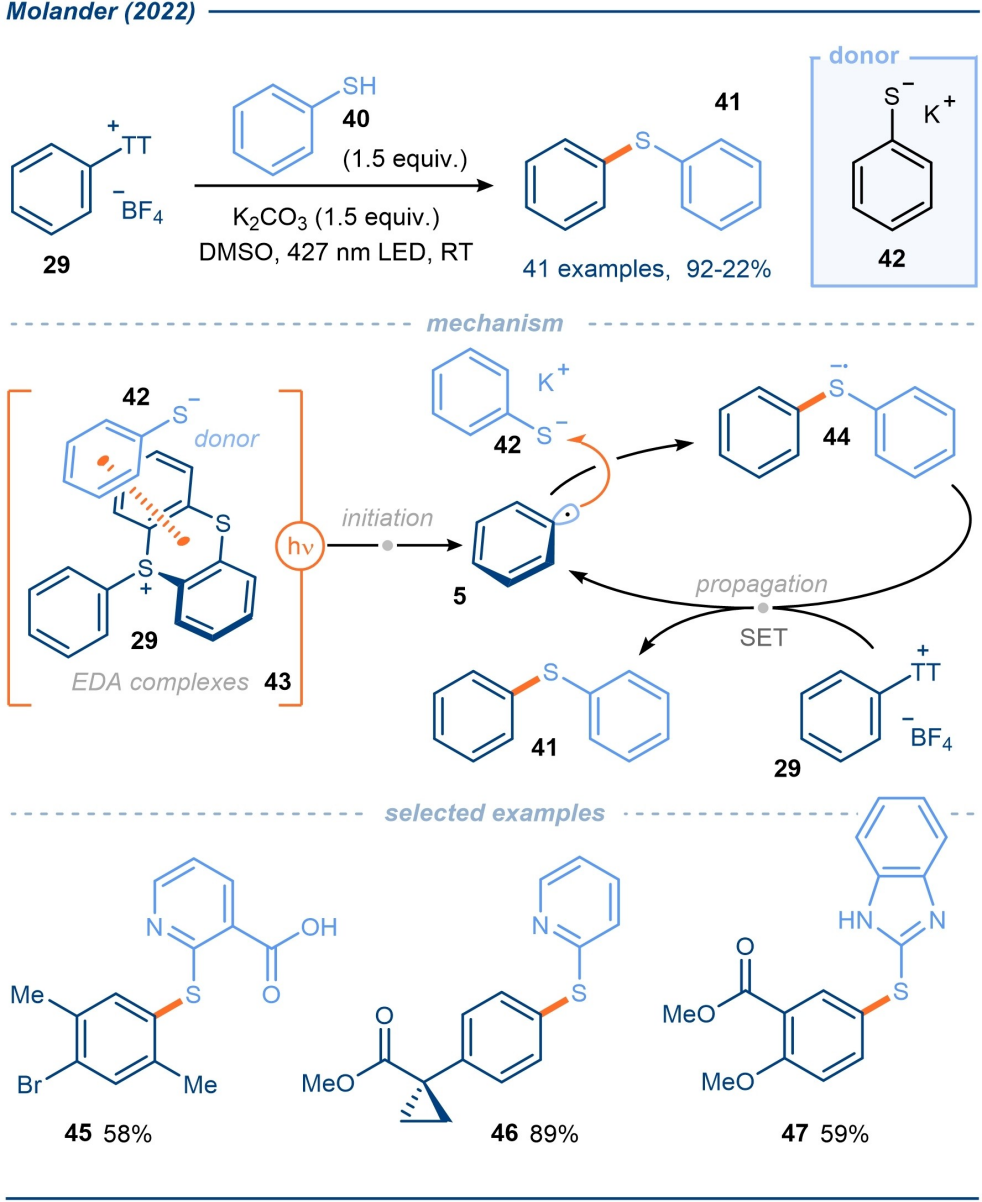
Exploitation of aryl sulfonium salts in EDA complexes with thiolate donors; application in aryl radical thiolation.^[23]^

Early in 2022, Molander and co‐workers reported a photoactivated coupling of thianthrenium salts **29** with thiophenols **40** to afford an array of biaryl sulfides **41** and that exploits EDA complexes **43** (Scheme 6).^[23]^ Miyake and co‐workers previously reported a related C(sp^2^)−S coupling in 2017,^[10c]^ using thiolates **42** as donors but using aryl halides as the acceptors in EDA complexes. For reasons discussed earlier, this method was mostly limited to the use of aryl halides bearing electron‐withdrawing groups, however, aryl halides without electron‐withdrawing groups, such as iodobenzene and iodotoluene, were shown to react given longer reaction times. By using thianthrenium salts **29**, Molander was able to expand the scope of biaryl sulfide formation to include a wide range of electron‐rich aryl radical sources including those containing reactive halides such as bromides and iodides. The ability to selectively cleave thianthrene and retain the halide handles represented a significant advance on earlier biaryl sulfide syntheses. Both light and base were required for the reaction, supporting the notion that thiolate **42** is the electron‐donating species in the reaction. Interestingly, the reaction was not perturbed by the presence of O_2_ and hence could be performed open to air. The authors reported a quantum yield for the reaction of 89 and therefore, in accordance with earlier work,[^10c^, ^24a^, ^24b^] they proposed a radical chain dominated mechanism (Scheme 6); following SET within EDA complexes **43**, the resulting aryl radicals **5** escape the solvent cage and couple with thiolate **42** affording radical anions **44**. These radical anions can propagate the chain by reducing thianthrenium salt **29** and concomitantly forming the coupled products **41**.

**Scheme 7:**
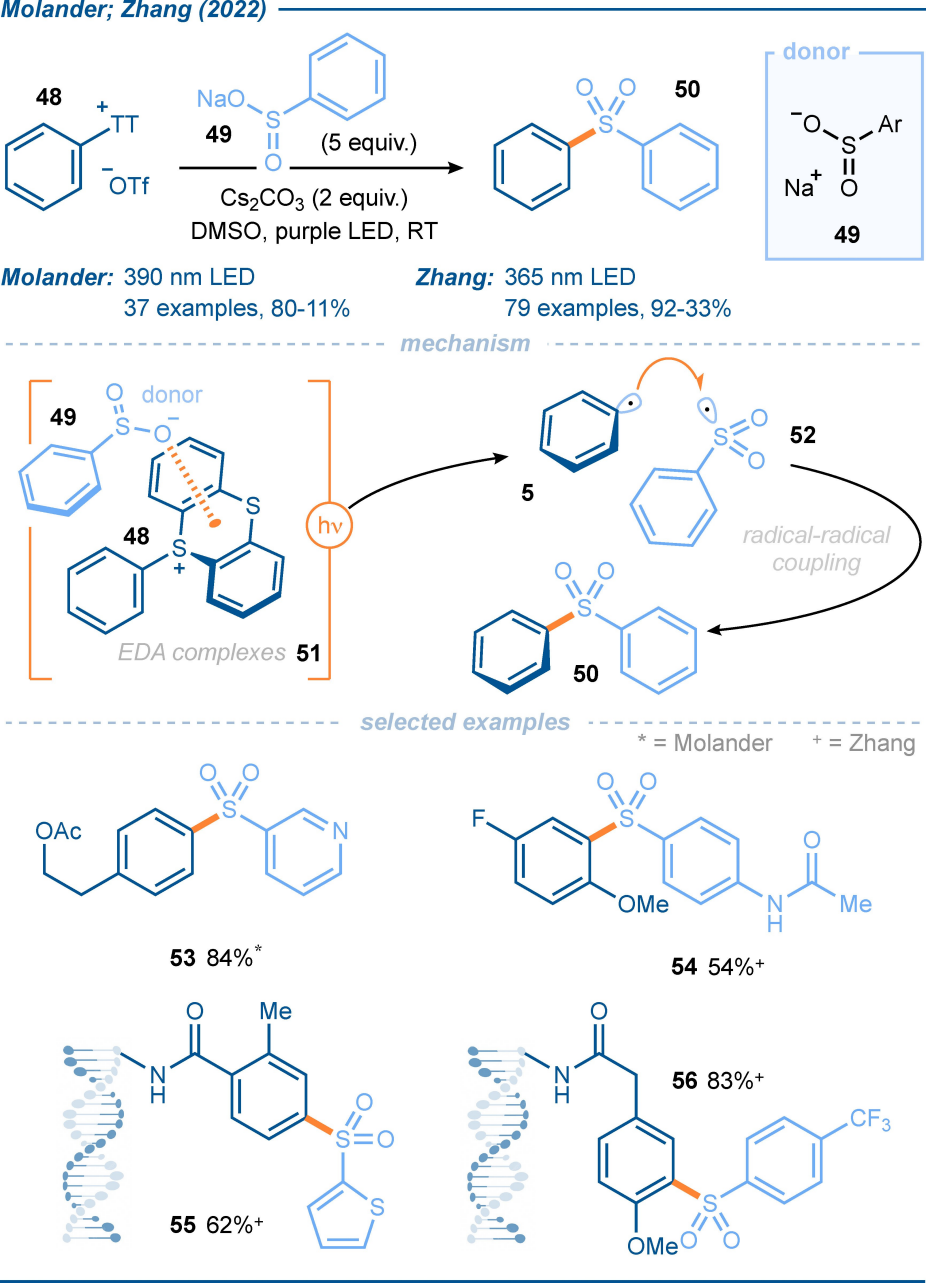
Exploitation of aryl sulfonium salts in EDA complexes with sulfinate donors; application in aryl radical sulfonylation.[^25^, ^26^]

Molander and co‐workers followed up on this work by developing a C(sp^2^)−S bond forming reaction, that delivers biaryl sulfones **50**, rather than sulfides (Scheme 7).^[25]^ A related process was published by Zhang and co‐workers later in the year.^[26]^ Both groups utilized aryl sodium sulfinates **49** as donors with thianthrenium salts **48** as acceptors in EDA complexes **51**. The approach afforded a large range of biaryl sulfones **50** and the late‐stage sulfonation of bioactive molecules was used to exemplify the selective nature of the process.^[26]^ The fact that light was essential for reactivity, combined with other mechanistic observations, supported a radical pathway rather than a S_N_Ar‐type pathway involving the nucleophilic sulfinates. Following SET upon photoexcitation of EDA complexes **51**, the resultant aryl radical **5** and sulfonyl radical **52** combine to afford sulfone products **50**. A range of light sources was shown to be effective in the reaction, however, the use of 365 nm provided the highest yield for Zhang (and 390 nm for Molander), while using green light (525 nm) gave the desired product in trace amounts only. The use of electron‐rich, neutral and electron‐poor aryl radical precursors all resulted in moderate to excellent yields, with proto‐dethianthrenation being noted as the main side reaction in most cases. A range of electron‐rich and electron‐poor aryl and heteroaryl sulfinates worked well in the reaction. Furthermore, in line with previous work from their group and others,^[27]^ Zhang expanded the application of this EDA complex approach to the modification of DNA encoded molecules (e.g. to give **55**, **56**) through a reversible adsorption to solid support (RASS) strategy, with the intention of broadening the structural diversity of the DNA‐encoded library (DEL). In some of the more complex on‐DNA reactions, the addition of water was found to improve the yield of product.

**Scheme 8:**
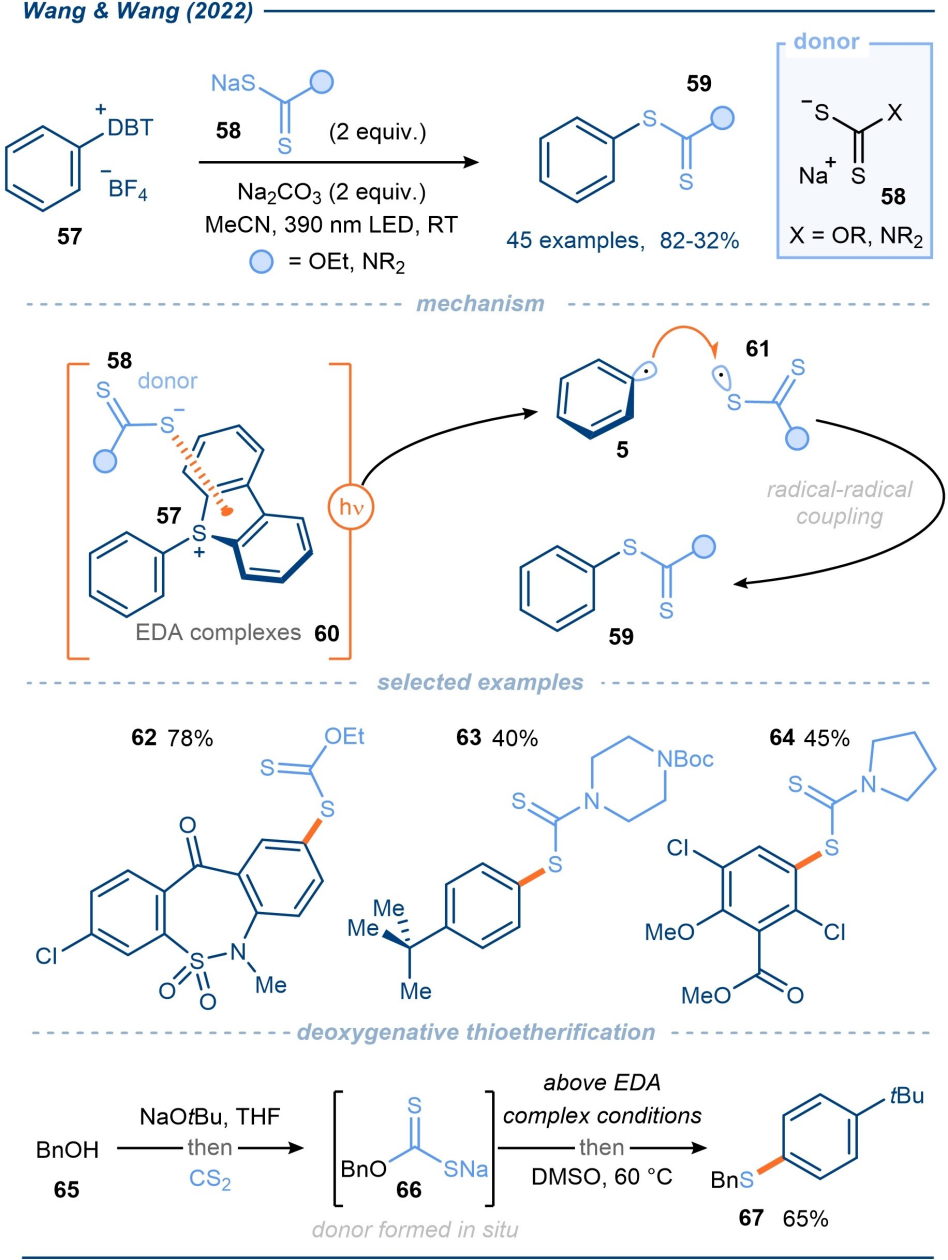
Exploitation of aryl sulfonium salts in EDA complexes with dithioate donors; application in the synthesis of aryl xanthates and aryl dithiocarbamates.^[28]^

Wang, Wang and co‐workers subsequently extended the scope of C(sp^2^)−S couplings by utilizing aryl sulfonium salts **57** as electron‐acceptors in EDA complexes **60** (Scheme 8).^[28]^ Their procedure employed two related classes of donor **58**; sodium ethyl xanthogenate,^[29]^ to afford aryl xanthates (e.g. **62**) (analogous to the Leuckart thiophenol reaction),^[30]^ and a variety of dithiocarbamate salts to afford the corresponding aryl dithiocarbamates (e.g. **63**–**64**). The reaction performed best when using 390 nm light, however, reactivity was also observed with longer wavelengths. In contrast to previous C(sp^2^)−S couplings, dibenzothiophenium salts were employed in the reaction. Halides (F, Cl, Br, I) were stable to the reaction conditions, again displaying the greater propensity for reductive cleavage of the C−S bond vs C−Hal bonds. The authors also proposed a radical‐radical coupling mechanism, whereby aryl radicals **5** coupled with radicals **61**, both formed upon SET from the donors **58** to the acceptors **57** in EDA complexes **60**. Impressively, deoxygenative thioetherification reactions of alkyl alcohols were possible by adapting the method; donor **66** was formed in situ from alcohol **65** and then employed in the EDA complex reaction, the subsequent aryl xanthates then fragmented to afford sulfide **67** upon exposure to heat with expulsion of carbonyl sulfide.

### 2.3 C(sp^2^)−B bond‐forming reactions

**Scheme 9:**
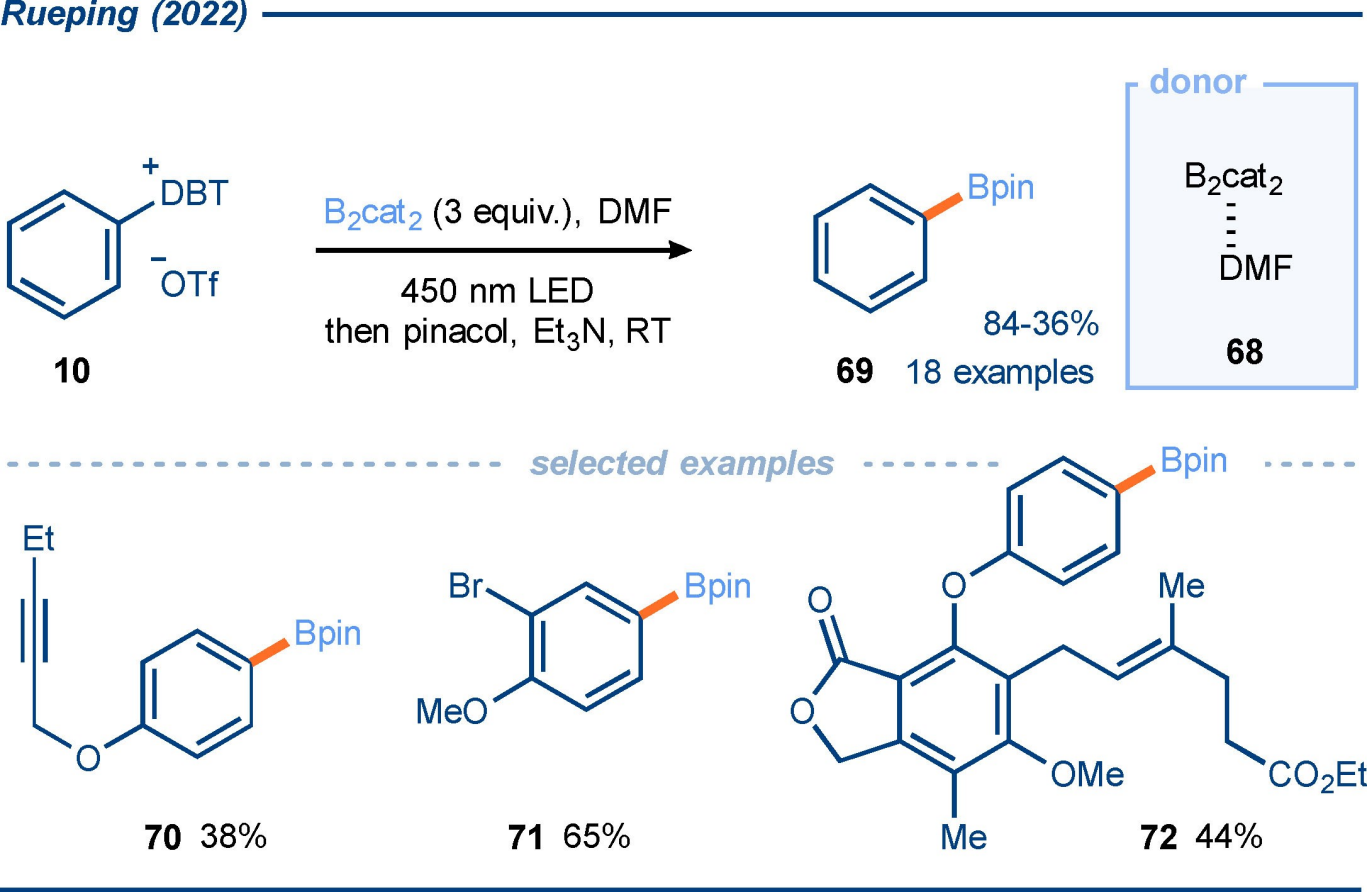
Exploitation of aryl sulfonium salts in EDA complexes with a B_2_cat_2_⋅DMF adduct as donor; application in aryl radical borylation.^[31]^

**Scheme 10:**
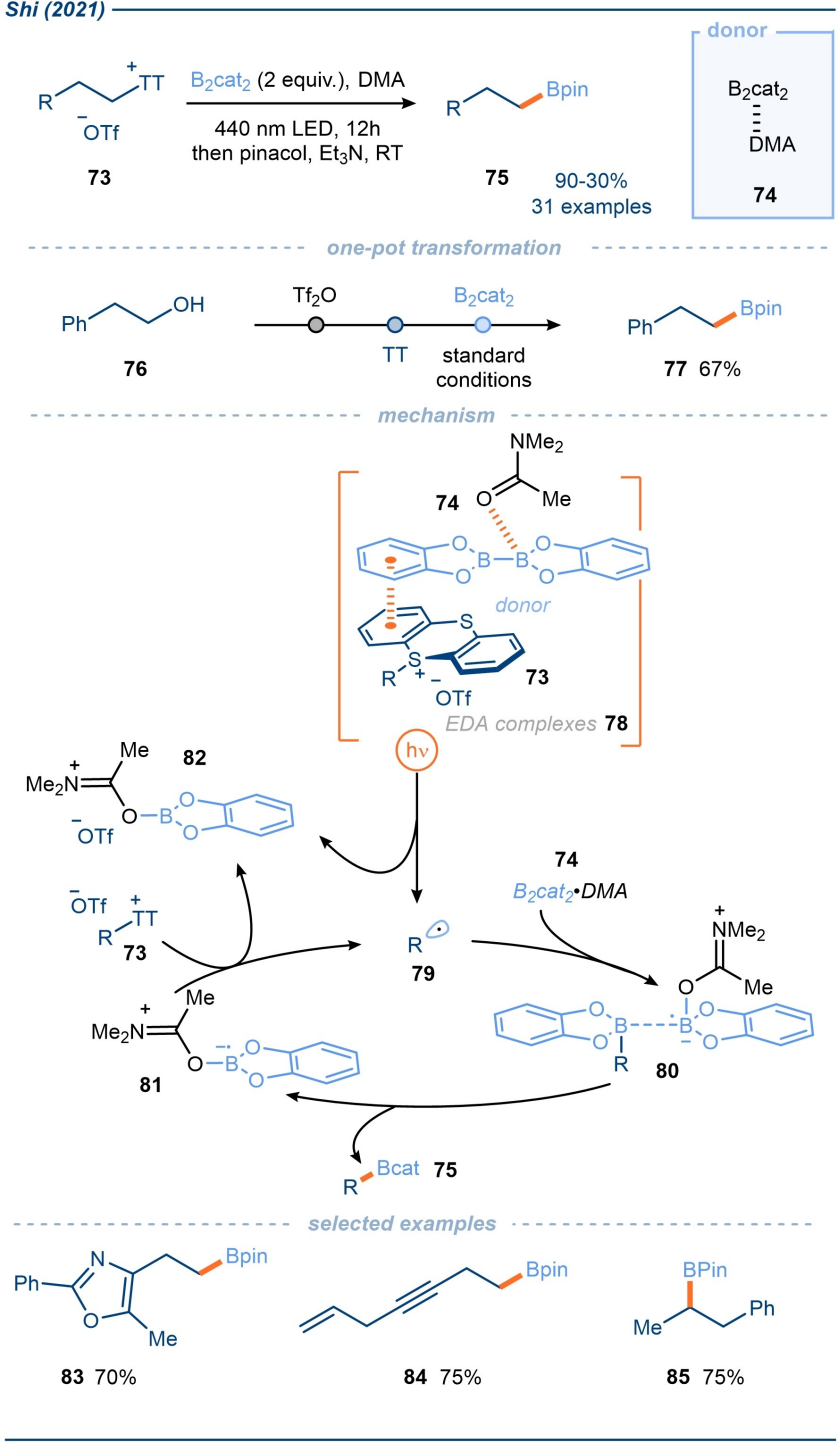
Exploitation of alkyl sulfonium salts in EDA complexes with a B_2_cat_2_⋅DMA adduct as donor; application in alkyl radical borylation.^[33]^ DMA=Dimethylacetamide.

In late 2022, Rueping and co‐workers reported the EDA complex mediated formation of aryl boronates **69** from the corresponding aryl dibenzothiophenium salts **10** and B_2_cat_2_ (Scheme 9).^[31]^ B_2_cat_2_ has been previously utilized as a donor in EDA complex mediated borylation reactions,[^12a^, ^32^] including by Shi and co‐workers for the borylation of alkyl radicals generated from sulfonium salts (see section 3.1)^[33]^ and by Studer^[34]^ for the borylation of aryl radicals generated by EDA complex chemistry. Using the predictable site selectivity of sulfonium salt formation by C−H functionalization, aryl boronates were selectively generated in good yield. Interestingly, using water as an additive was found to have a positive effect on the reaction, with 15 equivalents being reported as optimal. Much like earlier aryl radical generation methods utilizing EDA complexes of sulfonium salts, sensitive functionality was well tolerated by the reaction, including halides and unprotected hydroxyl groups. A mechanism analogous to that proposed by Shi (see Scheme 10) and Studer was invoked.

## 3 Alkyl radical generation

Alkyl radicals have been generated from sulfonium salts under a range of photochemical conditions. However, until recently, alkyl radical generation from sulfonium salts via EDA complexes was limited to the generation of trifluoromethyl radicals from Umemoto's reagent.^[15]^

Alkyl radical generation via EDA complex formation involving acceptors derived from native functionality other than sulfonium salts is well known; for example, amines derivatized as Katritzky salts[^35a^, ^35b^] and carboxylic acids activated as *N‐*hydroxyphthalimide esters.^[36]^ However, the reduction potential of alkyl sulfonium salts has been reported to be lower than these alternative alkyl radical generating EDA acceptors, and hence superior or new reactivity could well be identified using alkyl sulfonium salts.

### 3.1 C(sp^3^)−B bond‐forming reactions

In 2021, Shi and co‐workers reported the synthesis of primary and secondary alkyl boronates **75** from the corresponding alcohols, via formation of intermediate thianthrenium sulfonium salts **73** and subsequent activation through EDA complex formation (Scheme 10).^[33]^ Primary thianthrenium salt substrates **73** were synthesised by activation of the corresponding primary alcohols with Tf_2_O and subsequent substitution with thianthrene. Secondary alcohols were activated by conversion to the corresponding alkyl formates, through reaction with ethyl formate and Bi(OTf)_3_, followed by substitution with thianthrene. These salts were shown to form EDA complexes with a B_2_cat_2_⋅DMA adduct **74**, which upon photoactivation resulted in borylation of the generated alkyl radical **79**.

A range of alkyl sulfonium salts was tested in the borylation reaction. Those generated from tetrahydrothiophene—rather than thianthrene—afforded no product, supporting the notion that an aryl‐containing sulfonium salt is required for EDA complexation. Sulfonium salts derived from diphenyl sulfide suffered from the formation of phenyl‐borylation side products rather than the desired alkyl‐borylated products. Although sulfonium salts derived from DBT were proficient in the borylation reaction, the use of thianthrenium salts was shown to be optimal. Impressively, the authors displayed the viability of a one‐pot procedure, whereby alcohol **76** was converted into the sulfonium salt, followed by borylation under the photochemical conditions, affording product **77** in good overall yield. Although some functionality was not well tolerated under the sulfonium salt formation conditions (e.g. alkyl chlorides), halides were generally compatible with the subsequent borylation procedure. Additionally, the authors reported straightforward isolation of the thianthrene at the end of the reaction, highlighting the recyclability of sulfide starting materials in these sulfonium salt‐EDA complex approaches.

A quantum yield of 46, strongly indicated the operation of a radical chain process initiated by the photoexcitation of EDA complexes **78**, derived from the sulfonium salts **73** and B_2_cat_2_⋅DMA adduct **74** (Scheme 10). Irradiation initiated SET, forming the corresponding alkyl radical **79** following the fragmentation of the C−S bond. Radical **79** can then add to another B_2_cat_2_⋅DMA adduct **74**, affording radical complex **80**. Subsequent cleavage of the B−B bond results in the generation of the desired alkyl borylated products **75** and boryl radical **81**, which can then propagate the radical chain by reducing sulfonium salts **73**. Beyond the photo‐mediated process, the group also reported a thermal activation mode, although product yields were typically lower using this alternative approach.

## 4 Conclusion and Outlook

While sulfonium salts have been widely‐used in organic synthesis over the years, new ways of exploiting their reactivity and activation modes have led to them emerging as next generation coupling partners in a range of metal‐catalyzed and metal‐free processes.

Although sulfonium salt EDA complexes were known, only within the last two years has their full potential begun to be explored. However, many areas of sulfonium salt EDA complex chemistry remain undeveloped. For example, an outstanding feature of sulfonium salt chemistry is the efficient and selective methods available for their formation from unsaturated species. If this ease of access could be further developed and translated to other feedstocks, it would reduce the reliance on prefunctionalized partners that currently underpins known EDA complex chemistry. Additional challenges for the future include; extending the range of donors in sulfonium salt EDA complex chemistry; further engineering the sulfur‐containing units in sulfonium salt acceptors; rationally designing the donor and sulfur‐containing unit to suit a particular proposed transformation; using computational chemistry to understand the nature of sulfonium salt EDA complex chemistry and expedite process development, and finally; rendering the processes catalytic in the sulfur‐containing unit and thus further improving the sustainability of synthesis using sulfonium salt EDA complex chemistry.

In summary, sulfonium salts appear tailor‐made for exploitation in EDA complex‐mediated chemistry; their accessibility, stability, and high tuneability promises a versatility that will lend itself to the future development of general processes of broad synthetic scope.

## Conflict of interest

The authors declare no conflict of interest.

## Biographical Information

*Leendert van Dalsen obtained his B.A. in Chemistry from Trinity College Dublin in 2019, carrying out a final year research project on azide mediated γ‐lactam synthesis. In 2021, he joined the group of Prof. David J. Procter for his Ph.D. studies. His current research endeavours are focussed on the development and application of sulfonium salts*.

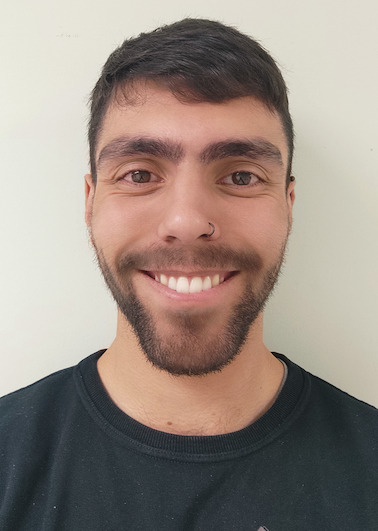

## Biographical Information

*Rachel E. Brown received her M.Chem. degree at the University of York in 2021, carrying out a final year research project in the diastereoselective synthesis of trans‐β‐aryl 5‐membered cyclic sulfoximines. In 2021, she joined the group of Prof. David J. Procter for her Ph.D. studies funded by the EPSRC and AstraZeneca. Her research aims to develop cross‐coupling reactions involving sulfonium salts*.

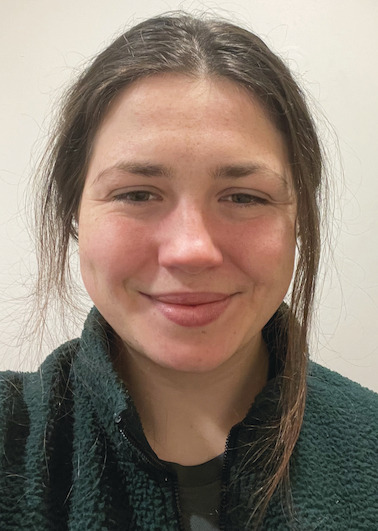

## Biographical Information

*James A. Rossi‐Ashton completed his Ph.D. studies in 2019 at the University of York under the supervision of Prof. Richard Taylor and Dr. William Unsworth. During his Ph.D., he was also a visiting scholar at the Shanghai Institute of Organic Chemistry working under Prof. ShuLi You. After roles as a Post‐Doctoral Research Associate with Prof. David J. Procter at the University of Manchester and with Prof. Dave MacMillan at Princeton University, he is now a Lecturer of Organic Chemistry at the University of Manchester*.

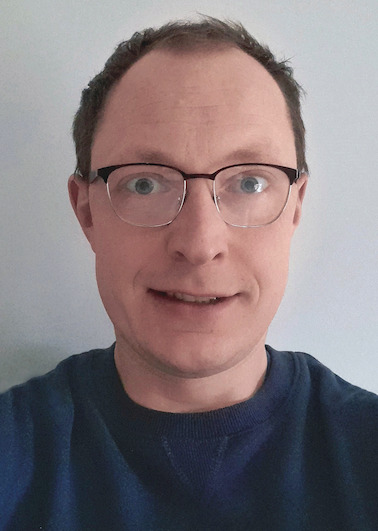

## Biographical Information

*David J. Procter was born in Leyland in Lancashire, England. He obtained his B.Sc. in chemistry from the University of Leeds in 1992 and his Ph.D. in 1995 working with Prof. Christopher Rayner. He then spent two years as a Post‐Doctoral Research Associate with Prof. Robert Holton at Florida State University in Tallahassee, USA, working on Taxol. In late 1997 he took up a Lectureship at the University of Glasgow in Scotland and was promoted to Senior Lecturer in 2004. In 2004, he moved to a Readership at the University of Manchester. David was promoted to Professor in 2008*.

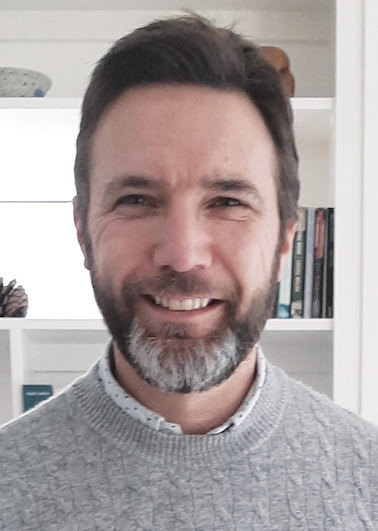

## References

[1] M. Yan , J. C. Lo , J. T. Edwards , P. S. Baran , J. Am. Chem. Soc. 2016, 138, 12692–12714. 27631602 10.1021/jacs.6b08856 PMC5054485

[2a] D. A. DiRocco , K. Dykstra , S. Krska , P. Vachal , D. V. Conway , M. Tudge , Angew. Chem. Int. Ed. 2014, 53, 4802–4806; 10.1002/anie.201402023 24677697

[2b] A. ElMarrouni , C. B. Ritts , J. Balsells , Chem. Sci. 2018, 9, 6639–6646. 30310596 10.1039/c8sc02253d PMC6115631

[3a] N. A. Romero , D. A. Nicewicz , Chem. Rev. 2016, 116, 10075–10166; 27285582 10.1021/acs.chemrev.6b00057

[3b] M. H. Shaw , J. Twilton , D. W. C. MacMillan , J. Org. Chem. 2016, 81, 6898–6926; 27477076 10.1021/acs.joc.6b01449 PMC4994065

[3c] J. A. Rossi-Ashton , A. K. Clarke , W. P. Unsworth , R. J. K. Taylor , ACS Catal. 2020, 10, 7250–7261; 32905246 10.1021/acscatal.0c01923 PMC7469205

[3d] J. D. Bell , J. A. Murphy , Chem. Soc. Rev. 2021, 50, 9540–9685; 34309610 10.1039/d1cs00311a

[3e] A. Y. Chan , I. B. Perry , N. B. Bissonnette , B. F. Buksh , G. A. Edwards , L. I. Frye , O. L. Garry , M. N. Lavagnino , B. X. Li , Y. Liang , E. Mao , A. Millet , J. V. Oakley , N. L. Reed , H. A. Sakai , C. P. Seath , D. W. C. MacMillan , Chem. Rev. 2022, 122, 1485–1542. 34793128 10.1021/acs.chemrev.1c00383 PMC12232520

[4a] R. Foster , J. Phys. Chem. 1980, 84, 2135–2141;

[4b] S. V. Rosokha , J. K. Kochi , Acc. Chem. Res. 2008, 41, 641–653; 18380446 10.1021/ar700256a

[4c] C. G. S. Lima , T. de M Lima , M. Duarte , I. D. Jurberg , M. W. Paixão , ACS Catal. 2016, 6, 1389–1407.

[5a] S. Zhou , T. Song , H. Chen , Z. Liu , H. Shen , C. Li , Org. Lett. 2017, 19, 698–701; 28102684 10.1021/acs.orglett.6b03870

[5b] R. P. Shirke , S. S. V. Ramasastry , Org. Lett. 2017, 19, 5482–5485; 28949540 10.1021/acs.orglett.7b02861

[5c] J. Hu , G. Wang , S. Li , Z. Shi , Angew. Chem. Int. Ed. 2018, 57, 15227–15231. 10.1002/anie.201809608 30253022

[6a] R. S. Mulliken , J. Am. Chem. Soc. 1952, 74, 811–824;

[6b] R. S. Mulliken , J. Phys. Chem. 1952, 56, 801–822;

[6c] E. F. Hilinski , J. M. Masnovi , C. Amatore , J. K. Kochi , P. M. Rentzepis , J. Am. Chem. Soc. 1983, 105, 6167–6168;

[6d] G. E. M. Crisenza , D. Mazzarella , P. Melchiorre , J. Am. Chem. Soc. 2020, 142, 5461–5476. 32134647 10.1021/jacs.0c01416 PMC7099579

[7a] D. Cantacuzène , C. Wakselman , R. Dorme , J. Chem. Soc. Perkin Trans. 1 1977, 1365–1371;

[7b] J. F. Bunnett , Acc. Chem. Res. 1978, 11, 413–420;

[7c] M. A. Fox , J. Younathan , G. E. Fryxell , J. Org. Chem. 1983, 48, 3109–3112;

[7d] P. A. Wade , H. A. Morrison , N. Kornblum , J. Org. Chem. 1987, 52, 3102–3107;

[7e] S. Sankararaman , W. A. Haney , J. K. Kochi , J. Am. Chem. Soc. 1987, 109, 7824–7838;

[7f] G. A. Russell , K. Wang , J. Org. Chem. 1991, 56, 3475–3479.

[8a] I. Kim , S. Park , S. Hong , Org. Lett. 2020, 22, 8730–8734; 33104358 10.1021/acs.orglett.0c03347

[8b] A. Lipp , S. O. Badir , R. Dykstra , O. Gutierrez , G. A. Molander , Adv. Synth. Catal. 2021, 363, 3507–3520. 35273472 10.1002/adsc.202100469 PMC8903066

[9] W. Zhou , S. Wu , P. Melchiorre , J. Am. Chem. Soc. 2022, 144, 8914–8919. 35549337 10.1021/jacs.2c03546

[10a] M. Tobisu , T. Furukawa , N. Chatani , Chem. Lett. 2013, 42, 1203–1205;

[10b] E. Arceo , I. D. Jurberg , A. Álvarez-Fernández , P. Melchiorre , Nat. Chem. 2013, 5, 750–756; 23965676 10.1038/nchem.1727

[10c] B. Liu , C.-H. Lim , G. M. Miyake , J. Am. Chem. Soc. 2017, 139, 13616–13619. 28910097 10.1021/jacs.7b07390 PMC5920654

[11] J. Davies , S. G. Booth , S. Essafi , R. A. W. Dryfe , D. Leonori , Angew. Chem. Int. Ed. 2015, 54, 14017–14021. 10.1002/anie.201507641 PMC4648045 26412046

[12a] A. Fawcett , J. Pradeilles , Y. Wang , T. Mutsuga , E. L. Myers , V. K. Aggarwal , Science 2017, 357, 283–286; 28619717 10.1126/science.aan3679

[12b] M.-C. Fu , R. Shang , B. Zhao , B. Wang , Y. Fu , Science 2019, 363, 1429–1434. 30923218 10.1126/science.aav3200

[13a] Z. Yang , Y. Liu , K. Cao , X. Zhang , H. Jiang , J. Li , Beilstein J. Org. Chem. 2021, 17, 771–799; 33889219 10.3762/bjoc.17.67 PMC8042489

[13b] T. Tasnim , M. J. Ayodele , S. P. Pitre , J. Org. Chem. 2022, 87, 10555–10563. 35904501 10.1021/acs.joc.2c01013

[14a] V. G. Nenaidenko , E. S. Balenkova , Russ. J. Org. Chem. 2003, 39, 291–330;

[14b] L. H. S. Smith , S. C. Coote , H. F. Sneddon , D. J. Procter , Angew. Chem. Int. Ed. 2010, 49, 5832–5844; 10.1002/anie.201000517 20583014

[14c] A. P. Pulis , D. J. Procter , Angew. Chem. Int. Ed. 2016, 55, 9842–9860; 10.1002/anie.201601540 27409984

[14d] D. Kaiser , I. Klose , R. Oost , J. Neuhaus , N. Maulide , Chem. Rev. 2019, 119, 8701–8780; 31243998 10.1021/acs.chemrev.9b00111 PMC6661881

[14e] S. I. Kozhushkov , M. Alcarazo , Eur. J. Inorg. Chem. 2020, 2020, 2486–2500, https://chemistry-europe.onlinelibrary.wiley.com/doi/abs/10.1002/ejic.202000249; 32742188 10.1002/ejic.202000249 PMC7386937

[14f] Á. Péter , G. J. P. Perry , D. J. Procter , Adv. Synth. Catal. 2020, 362, 2135–2142;

[14g] R. Fan , C. Tan , Y. Liu , Y. Wei , X. Zhao , X. Liu , J. Tan , H. Yoshida , Chin. Chem. Lett. 2021, 32, 299–312;

[14h] F. R. Berger , T. Ritter , Synlett 2021, 33, 339–345;

[14i] M. Mondal , S. Connolly , S. Chen , S. Mitra , N. J. Kerrigan , Organics 2022, 3, 320–363;

[14j] G. J. P. Perry , H. Yorimitsu , ACS Sustainable Chem. Eng. 2022, 10, 2569–2586.

[15a] Y. Macé , C. Pradet , M. Popkin , J.-C. Blazejewski , E. Magnier , Tetrahedron Lett. 2010, 51, 5388–5391;

[15b] Y. Cheng , X. Yuan , J. Ma , S. Yu , Chem. Eur. J. 2015, 21, 8355–8359; 25907421 10.1002/chem.201500896

[15c] H. Wang , Y. Cheng , S. Yu , Sci. China Chem. 2016, 59, 195–198;

[15d] M. L. Spell , K. Deveaux , C. G. Bresnahan , B. L. Bernard , W. Sheffield , R. Kumar , J. R. Ragains , Angew. Chem. Int. Ed. 2016, 55, 6515–6519; 10.1002/anie.201601566 27086646

[15e] M. Zhu , K. Zhou , X. Zhang , S.-L. You , Org. Lett. 2018, 20, 4379–4383. 29985618 10.1021/acs.orglett.8b01899

[16a] M. H. Aukland , M. Šiaučiulis , A. West , G. J. P. Perry , D. J. Procter , Nat. Catal. 2020, 3, 163–169;

[16b] J. Li , J. Chen , R. Sang , W.-S. Ham , M. B. Plutschack , F. Berger , S. Chabbra , A. Schnegg , C. Genicot , T. Ritter , Nat. Chem. 2020, 12, 56–62. 31767996 10.1038/s41557-019-0353-3

[17] N. Kvasovs , V. Gevorgyan , Chem. Soc. Rev. 2021, 50, 2244–2259. 33313618 10.1039/d0cs00589d PMC7920999

[18] I. Bosque , T. Bach , ACS Catal. 2019, 9, 9103–9109.

[19] X.-Q. Hu , Z.-K. Liu , Y.-X. Hou , Y. Gao , iScience 2020, 23, 101266. 32593954 10.1016/j.isci.2020.101266 PMC7327862

[20a] A. Dewanji , L. van Dalsen , J. Rossi-Ashton , E. Gasson , G. Crisenza , D. J. Procter , Nat. Chem. 2023, 15, 43–52; 36471045 10.1038/s41557-022-01092-y

[20b] A. Dewanji, L. van Dalsen, J. Rossi-Ashton, E. Gasson, G. Crisenza, D. J. Procter, *ChemRxiv* **2021**, 10.26434/chemrxiv-2021-xbn7k. 36471045

[21a] L. Marzo , S. Wang , B. König , Org. Lett. 2017, 19, 5976–5979; 29064719 10.1021/acs.orglett.7b03001

[21b] K. Liang , N. Li , Y. Zhang , T. Li , C. Xia , Chem. Sci. 2019, 10, 3049–3053. 30996886 10.1039/c8sc05170d PMC6427940

[22] K. Sun , A. Shi , Y. Liu , X. Chen , P. Xiang , X. Wang , L. Qu , B. Yu , Chem. Sci. 2022, 13, 5659–5666. 35694358 10.1039/d2sc01241c PMC9116284

[23] M. J. Cabrera-Afonso , A. Granados , G. A. Molander , Angew. Chem. Int. Ed. 2022, 61, e202202706, https://onlinelibrary.wiley.com/doi/abs/10.1002/anie.202202706. 10.1002/anie.202202706 PMC9117462 35294095

[24a] J. F. Bunnett , X. Creary , J. Org. Chem. 1974, 39, 3173–3174;

[24b] T. Uchikura , Y. Hara , K. Tsubono , T. Akiyama , ACS Org. Inorg. Au 2021, 1, 23–28. 36855634 10.1021/acsorginorgau.1c00007 PMC9954416

[25] A. Granados , M. J. Cabrera-Afonso , M. Escolano , S. O. Badir , G. A. Molander , Chem Catal. 2022, 2, 898–907. 35846835 10.1016/j.checat.2022.03.007 PMC9282721

[26] Y. Zhang , S. Xia , W. Shi , B. Lin , X. Su , W. Lu , X. Wu , X. Wang , X. Lu , M. Yan , X. Zhang , Org. Lett. 2022, 24, 7961–7966. 36278920 10.1021/acs.orglett.2c03077

[27a] J. P. Phelan , S. B. Lang , J. Sim , S. Berritt , A. J. Peat , K. Billings , L. Fan , G. A. Molander , J. Am. Chem. Soc. 2019, 141, 3723–3732; 30753065 10.1021/jacs.9b00669 PMC6393171

[27b] B. Lin , W. Lu , Z. Chen , Y. Zhang , Y. Duan , X. Lu , M. Yan , X. Zhang , Org. Lett. 2021, 23, 7381–7385; 34546064 10.1021/acs.orglett.1c02562

[27c] S. Patel , S. O. Badir , G. A. Molander , Trends Chem. 2021, 3, 161–175. 33987530 10.1016/j.trechm.2020.11.010 PMC8112611

[28] M. Zhang , B. Wang , Y. Cao , Y. Liu , Z. Wang , Q. Wang , Org. Lett. 2022, 24, 8895–8900. 36441902 10.1021/acs.orglett.2c03736

[29] E. de Pedro Beato , D. Spinnato , W. Zhou , P. Melchiorre , J. Am. Chem. Soc. 2021, 143, 12304–12314. 34320312 10.1021/jacs.1c05607 PMC8361436

[30] J. R. Cox, Jr. , C. L. Gladys , L. Field , D. E. Pearson , J. Org. Chem. 1960, 25, 1083–1092.

[31] B. Li , K. Wang , H. Yue , A. Drichel , J. Lin , Z. Su , M. Rueping , Org. Lett. 2022, 24, 7434–7439. 36191259 10.1021/acs.orglett.2c03008

[32] J. Wu , L. He , A. Noble , V. K. Aggarwal , J. Am. Chem. Soc. 2018, 140, 10700–10704. 30091912 10.1021/jacs.8b07103

[33] C. Chen , Z.-J. Wang , H. Lu , Y. Zhao , Z. Shi , Nat. Commun. 2021, 12, 4526, https://www.nature.com/articles/s41467-021-24716-2. 34312381 10.1038/s41467-021-24716-2 PMC8313578

[34] Y. Cheng , C. Mück-Lichtenfeld , A. Studer , Angew. Chem. Int. Ed. 2018, 57, 16832–16836. 10.1002/anie.201810782 PMC6470957 30332527

[35a] J. Wu , P. S. Grant , X. Li , A. Noble , V. K. Aggarwal , Angew. Chem. Int. Ed. 2019, 58, 5697–5701; 10.1002/anie.201814452 PMC6492299 30794331

[35b] M. Yang , T. Cao , T. Xu , S. Liao , Org. Lett. 2019, 21, 8673–8678. 31638821 10.1021/acs.orglett.9b03284

[36] S. He , H. Li , X. Chen , I. B. Krylov , A. O. Terent'ev , L. Qu , B. Yu , Chin. J. Org. Chem. 2021, 41, 4661–4689.

